# Probiotic Bacteria Regulate Intestinal Epithelial Permeability in Experimental Ileitis by a TNF-Dependent Mechanism

**DOI:** 10.1371/journal.pone.0042067

**Published:** 2012-07-25

**Authors:** Daniele Corridoni, Luca Pastorelli, Benedetta Mattioli, Silviu Locovei, Dai Ishikawa, Kristen O. Arseneau, Marcello Chieppa, Fabio Cominelli, Theresa T. Pizarro

**Affiliations:** 1 Department of Pathology, Case Western Reserve University School of Medicine, Cleveland, Ohio, United States of America; 2 Division of Gastroenterology and Liver Disease, Department of Medicine, Case Western Reserve University School of Medicine, Cleveland, Ohio, United States of America; 3 Lab of Experimental Immunopathology, Instituto di Ricovero e Cura a Carattere Scientifico (IRCCS) “De Bellis”, Castellana Grotte, Bari, Italy; University of Chicago, United States of America

## Abstract

**Background:**

We previously showed that the probiotic mixture, VSL#3, prevents the onset of ileitis in SAMP/YitFc (SAMP) mice, and this effect was associated with stimulation of epithelial-derived TNF. The aim of this study was to determine the mechanism(s) of VSL#3-mediated protection on epithelial barrier function and to further investigate the “paradoxical” effects of TNF in preventing SAMP ileitis.

**Methods:**

Permeability was evaluated in SAMP mice prior to the onset of inflammation and during established disease by measuring transepithelial electrical resistance (TEER) on *ex vivo*-cultured ilea following exposure to VSL#3 conditioned media (CM), TNF or VSL#3-CM + anti-TNF. Tight junction (TJ) proteins were assessed by qRT-PCR, Western blot, and confocal microscopy, and TNFRI/TNFRII expression measured in freshly isolated intestinal epithelial cells (IEC) from SAMP and control AKR mice.

**Results:**

Culture with either VSL#3-CM or TNF resulted in decreased ileal paracellular permeability in pre-inflamed SAMP, but not SAMP with established disease, while addition of anti-TNF abrogated these effects. Modulation of the TJ proteins, claudin-2 and occludin, occurred with a significant decrease in claudin-2 and increase in occludin following stimulation with VSL#3-CM or TNF. TNF protein levels increased in supernatants of SAMP ilea incubated with VSL#3-CM compared to vehicle, while IEC-derived TNFR mRNA expression decreased in young, and was elevated in inflamed, SAMP versus AKR mice.

**Conclusions:**

Our data demonstrate that the previously established efficacy of VSL#3 in preventing SAMP ileitis is due to direct innate and homeostatic effects of TNF on the gut epithelium, modulation of the TJ proteins, claudin-2 and occludin, and overall improvement of intestinal permeability.

## Introduction

Inflammatory bowel disease (IBD) is a chronic relapsing inflammatory disorder of the gastrointestinal tract that includes Crohn’s disease (CD) and ulcerative colitis (UC). Although the precise etiology is currently unknown, it is generally accepted that IBD results from dysregulated immune responses to environmental factors in genetically susceptible individuals. As early as 1972, Shorter *et al*. proposed the hypothesis that the primary defect in CD may be due to an abnormal gut epithelial barrier, and further stipulated that compromised barrier function allows for increased passage of antigen(s) across the intestinal mucosa, resulting in an overactive immune response and chronic inflammation [1]. Further support of this concept comes from studies demonstrating that IBD patients display: 1) increased intestinal epithelial permeability versus control subjects, 2) disrupted barrier function that is not isolated to sites of active inflammation, and 3) first-degree relatives, as well as CD patients prior to disease relapse, possess increased gut permeability [2]–[6]. Together, these data suggest that patients with IBD have abnormally high gastrointestinal epithelial permeability and barrier dysfunction that may be a predisposing factor to chronic intestinal inflammation.

Similarly, SAMP1/YitFc (SAMP) mice display an inherent increase in small intestinal epithelial paracellular permeability that precedes the histologic onset of ileitis and is independent of commensal flora colonization [7], [8]. The SAMP strain represents a spontaneous model of chronic intestinal inflammation that resembles CD for disease location (*i.e*., terminal ileum), histologic features, and response to standard therapies used to treat Crohn’s patients [9]–[12]. Altered barrier function in the SAMP small intestine may stem from aberrant expression of claudin-2 and occludin that were found to be increased and decreased, respectively, compared to the control parental (AKR) strain [8]. In fact, increased claudin-2 and decreased occludin expression in epithelial tight junction (TJ) fibrils have been shown to generate weaker anastomoses between neighboring cells, resulting in barrier dysfunction [13]–[16]. These aforementioned trends in claudin-2 and occludin expression have also been reported in patients with both CD and UC [17], [18].

Recently, our group reported that administration of the probiotic formulation, VSL#3, prevents the onset of ileitis in SAMP mice [19]. VSL#3 is a mixture of *Streptococcus thermophilus*, three strains of *Bifidobacterium* and four strains of *Lactobacillus*, and has been proven to be efficacious in treating established colitis in IL-10 knockout (KO) mice [20]–[22], and to induce remission in patients with UC [23]–[27]. In SAMP mice, the beneficial effects of VSL#3 appear to be associated with increased production of epithelial-derived TNF and improved barrier function [19]. However, the mechanism(s) of VSL#3 modulation of intestinal permeability and the precise role of TNF in this process are not fully elucidated. The goal of the present study was to determine how VSL#3 functions to promote gut health through stimulation of epithelial innate immunity, and to define the direct role of early TNF expression in preventing SAMP ileitis.

We provide evidence, herein that recombinant TNF or VSL#3-stimulated TNF, specifically improves transepithelial permeability during the early phases of gut inflammation in young, pre-inflamed SAMP mice, by directly regulating the TJ proteins, claudin-2 and occludin and inducing innate, proinflammatory cytokine production. In addition, VSL#3-mediated changes in epithelial permeability appear to be dependent on decreased expression of TNFRs prior to the onset of ileitis. Our findings provide evidence that the beneficial effect of VSL#3 on intestinal permeability are directly mediated by a mechanism involving modulation of TJ proteins and TNF expression in the gut epithelium. Furthermore, our studies support the concept that TNF, in addition to its known proinflammatory activities, may also possess anti-inflammatory and homeostatic properties, particularly during the earlier phases of disease.

## Results

### VSL#3-CM and TNF Improve Inherent Epithelial Permeability Defect in Young SAMP Mice

**Figure 1:**
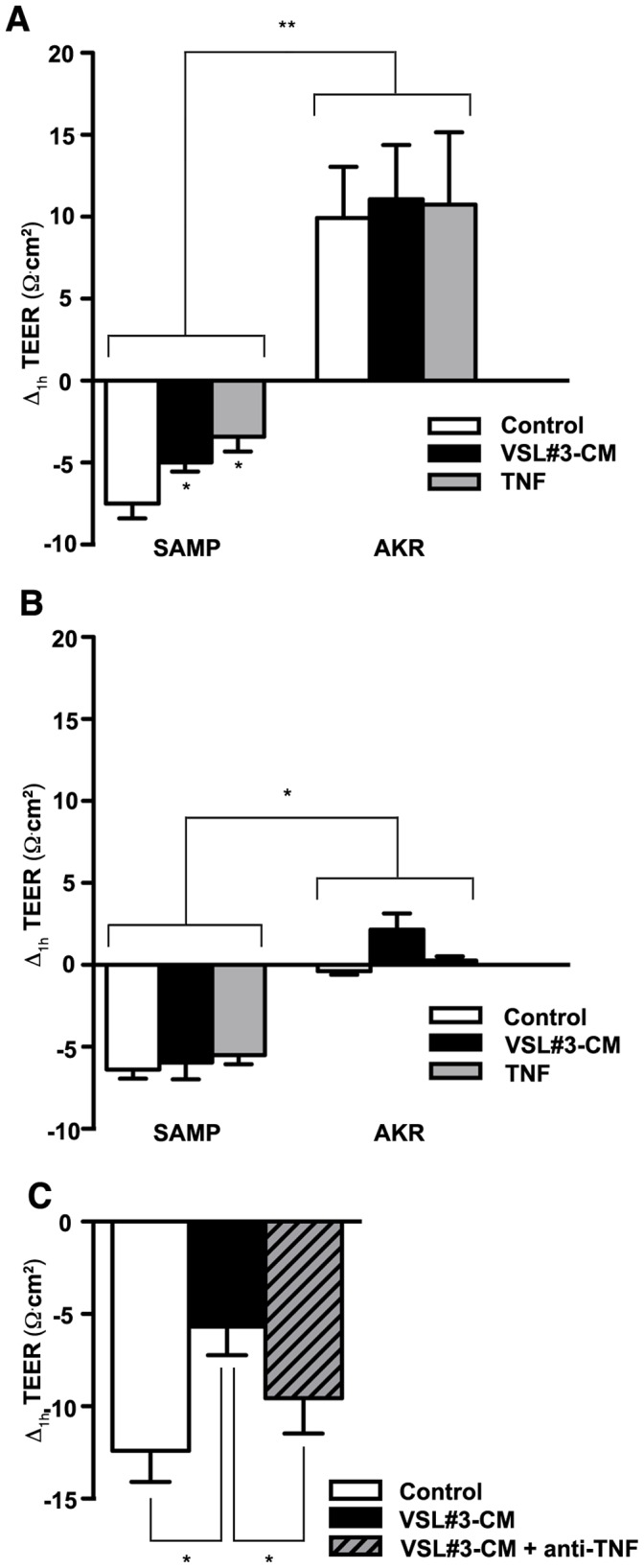
VSL#3 decreases epithelial paracellular permeability in a TNF-dependent manner on ilea from pre-inflamed SAMP mice. Epithelial paracellular permeability was assessed by measuring TEER on *ex vivo* cultured full-thickness ilea from (*A*), 3- to 4-wk old SAMP, prior to the onset of inflammation, (*B*), older (>20 wks) SAMP with established ileitis, and age-matched AKR controls at T_0_ and after 1 h (ΔTEER) exposure to VSL#3-CM or TNF (N = 6/exp grp). **p<0.01 vs. age- and treatment-matched AKR, *p<0.05 vs. vehicle cont. (*C*), Addition of a neutralizing antibody against TNF abrogated the positive effects of VSL#3 on ΔTEER in pre-inflamed ilea from young SAMP (N = 7 exp grp). *p<0.05 vs. vehicle cont. Results are combined from two independent experiments and expressed as mean ± SEM.

The effect of VSL#3-CM and TNF in regulating small intestinal barrier function was initially investigated by measuring *ex vivo* epithelial paracellular permeability in ilea from young SAMP mice, prior to the onset of inflammation (4-wk-old), SAMP with established disease (>20 weeks of age), and age-matched AKR controls (Fig. 1*A and B*, respectively) following treatment with either VSL#3-CM, TNF or vehicle control. Specifically, the change in TEER (ΔTEER) was measured in ilea after 1h exposure to VSL#3-CM or TNF versus vehicle control. Baseline levels (vehicle-treated) of 4- and >20-wk-old AKR ilea showed a decrease in epithelial paracellular permeability (increased TEER) during the incubation period due to tissue “normalization” under static conditions. In contrast, both 4- and >20-wk-old SAMP ilea showed an increased in epithelial paracellular permeability (decreased TEER), confirming the inherent defect in barrier function in SAMP mice previously reported by our group [7]. Importantly, exposure to either VSL#3-CM or TNF improved barrier function, and showed a decrease in ileal epithelial paracellular permeability (increased TEER) in young (*p*<0.05), but not old, SAMP mice (Fig. 1*A-B*). No significant differences were observed in the ΔTEER from AKR ilea, regardless of age, when exposed to either VSL#3-CM or TNF. In addition, to determine whether the effect of VSL#3-CM on improving epithelial barrier function in young SAMP mice may be directly mediated by TNF, ΔTEER was measured on 4-wk-old SAMP ilea cultured with VSL#3-CM in which a neutralizing antibody for TNF was added (Fig. 1*C*). In fact, anti-TNF had the ability to abrogate the effects of VSL#3-CM on epithelial paracellular permeability and markedly decreased ΔTEER compared to SAMP ilea exposed to VSL#3-CM alone (*p*<0.05). Taken together, these data indicate that both VSL#3-CM and TNF are efficacious in improving barrier function by decreasing epithelial paracellular permeability specifically in young SAMP that do not yet show overt histologic signs of ileal inflammation. In contrast, these effects are not observed in older SAMP mice with established disease or AKR controls, regardless of age. These observations are consistent with the *in vivo* setting wherein orally-administered VSL#3 to young non-inflamed SAMP, but not to older SAMP, effectively decreases epithelial permeability and prevents the onset of ileitis [19]. Our data further suggests that the effects of VSL#3 on epithelial barrier function may be directly mediated by TNF.

### VSL#3-CM Stimulates Production of Innate Mucosal Cytokines

**Figure 2:**
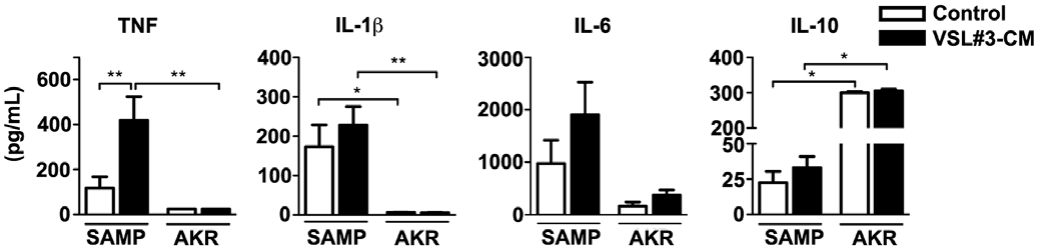
Increased ileal innate-type cytokine secretion following VSL3# treatment. Supernatants were collected from cultured ilea that were exposed apically to VSL#3-CM and cytokine protein levels measured as described in Methods and Materials. *p<0.05 and **p<0.01 vs. vehicle control or age- and treatment-matched AKR. Results are representative of two independent experiments (N = 6/exp grp) and expressed as mean ± SEM.

Emerging evidence suggests that defects in innate immunity and the inability of the gut mucosa to mount appropriate innate immune responses may result in the initiation and perpetuation of chronic intestinal inflammation, such as that observed in IBD [28], [29]. As such, we next tested whether probiotics are capable to enhance, and potentially “boost,” gut mucosal immunity through the production of innate-type cytokines in ileitis-prone SAMP mice. Full-thickness ilea from pre-inflamed, 4-wk-old SAMP and age-matched control AKR were cultured in the presence or absence of VSL#3 and cytokine production subsequently measured (Fig. 2). In support of our previous *in vivo* studies [19], secreted TNF protein levels increased after exposure to VLS#3-CM compared to vehicle control in SAMP mice (*p*<0.05). Moreover, other early, innate cytokines also showed a trend towards increased secretion following treatment with VSL#3-CM, including IL-1β and IL-6, with baseline levels of these cytokines greater than age-matched AKRs. Conversely, VSL#3-CM did not alter production of TNF or other innate-type cytokines from control AKR ilea. Interestingly, baseline IL-10 levels were markedly increased in AKR ilea compared to SAMP (*p*<0.01), but while VSL#3-CM had the ability to further induce IL-10 production from SAMP ilea (*p*<0.05), it did not modulate IL-10 in ilea from AKR mice. The effect of VSL#3-CM on inducing TNF secretion confirms TNF’s central role in VSL#3-mediated events, while the induction of other innate-type cytokines suggests that VSL#3 may play a role in augmenting global innate mucosal immune responses, including improving epithelial barrier function.

### VSL#3-CM and TNF Modulate TJ Protein Expression in Pre-inflamed, Young SAMP Mice

**Figure 3:**
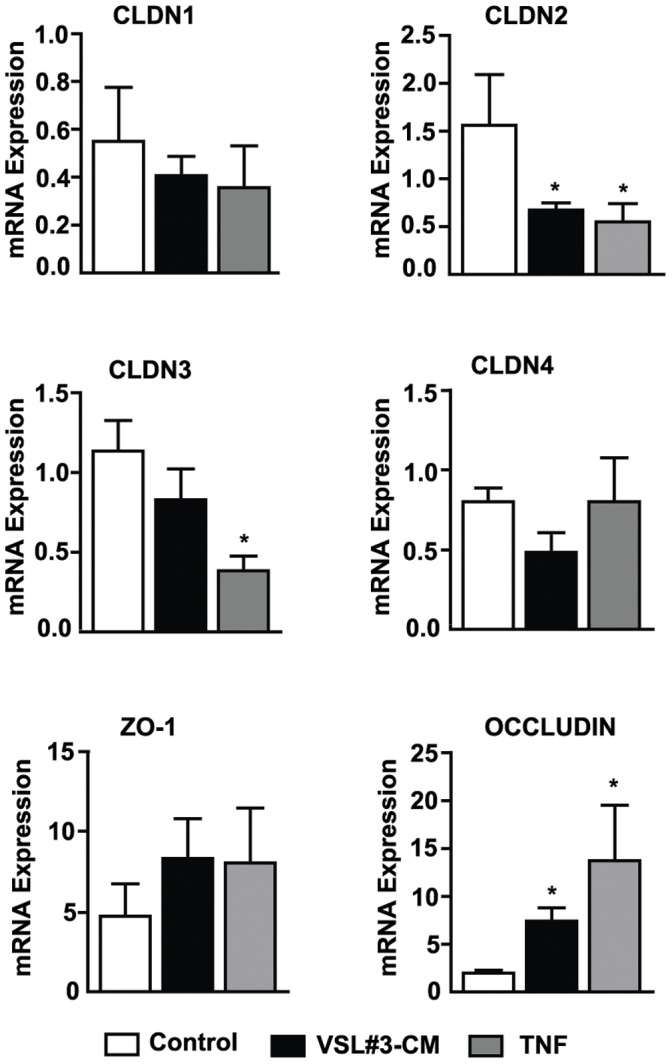
VSL#3 and TNF modulate TJ protein expression in ilea from pre-inflamed SAMP mice. mRNA transcript levels of TJ proteins (claudins 1–4, ZO-1, and occludin) were measured by qRT-PCR and expressed relative to ß-actin in full-thickness ilea from experimental mice following *ex vivo* culture with either VSL#3-CM or TNF. The greatest differences in expression were seen in claudin-2 (decrease) and occludin (increase) after either VSL#3-CM or TNF stimulation, and in claudin-3 (decrease) after TNF stimulation only. No differences in expression were noted for claudins 1, 4, and ZO-1 following exposure to either VSL#3-CM or TNF. *p<0.05 vs. vehicle cont. Results are representative of two independent experiments (N = 6/exp group) and expressed as mean ± SEM.

**Figure 4:**
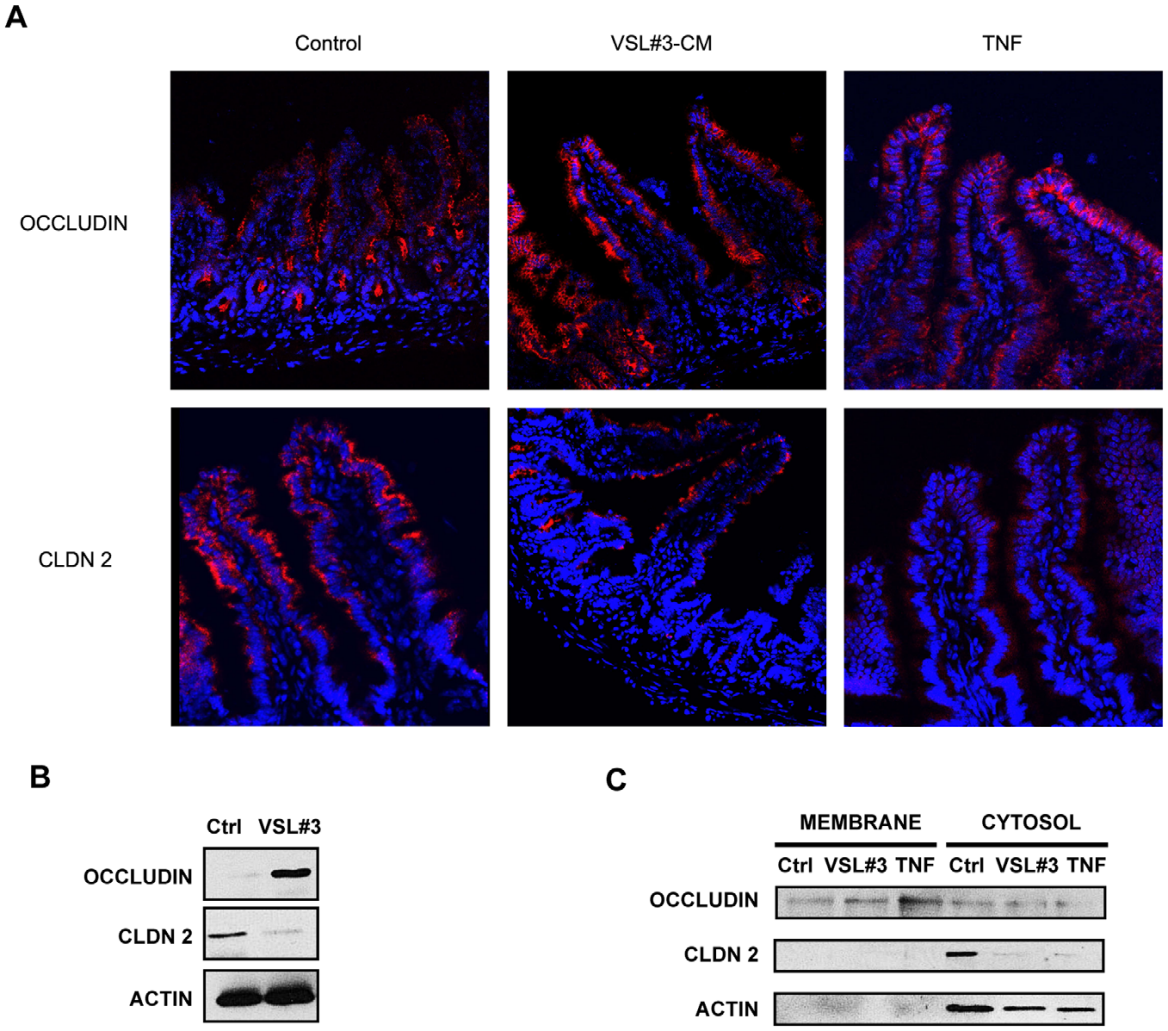
Localization and protein expression of epithelial claudin-2 and occludin following *in vivo* treatment with VSL#3-CM and TNF. (*A*), Representative confocal images (40X magnification) of ileal loops from young SAMP exposed to either VSL#3-CM, TNF or vehicle control, and immunofluorescent staining performed to detect occludin (upper panels) claudin-2 (lower panels). Epithelial occludin expression potently increased also in the villous tips after either VSL#3-CM or TNF stimulation in SAMP ilea. Claudin-2 primarily localized to the villous tips of SAMP ilea and decreased following VSL#3-CM, and particularly TNF, treatment. Results are representative of two independent experiments. (*B*), Representative Western blot of unfractionated IEC extracts isolated from SAMP mice treated with high dose VSL#3 showing increased expression of occludin and decreased claudin-2 protein levels compared to control. Results are representative of three independent experiments. (*C*), Representative Western blots demonstrating increased protein levels of occludin in membrane vs. cytosolic fractions of IEC from SAMP ileal loops treated with either VSL#3 or TNF compared to control. Claudin-2 protein was predominantly expressed in cytosolic vs. membrane fractions, and decreased following treatment with both VSL#3-CM and TNF compared to control. Results are representative of two independent experiments, pooling IEC from N = 3 mice/exp grp.

To elucidate potential mechanisms involved in VSL#3- and TNF-mediated improvement in epithelial barrier function and subsequent prevention of chronic intestinal inflammation in SAMP mice [19], we first studied TJ protein mRNA expression in full-thickness ilea from pre-inflamed SAMP following *ex vivo* organ culture with either VSL#3-CM or TNF (Fig. 3). Expression of claudins-1 and -4, and ZO-1 was not different following exposure to either VSL#3-CM or TNF compared to vehicle controls, while claudin-3 was significantly decreased after stimulation with TNF only (*p*<0.05). Interestingly, treatment with either VSL#3-CM or TNF, compared to vehicle, decreased claudin-2 and increased occludin mRNA levels (both *p*<0.05) in ilea from pre-inflamed SAMP mice. VSL#3- and TNF-specific modulation of claudin-2 and occludin in young, ileitis-prone SAMP mice was further confirmed by *in vivo* experiments on isolated ileal loops in which either VSL#3-CM or TNF was injected, followed by epithelial immunolocalization of the aforementioned TJ proteins (Fig. 4). Claudin-2 expression decreased, particularly in the distal tips of ilea following treatment with VSL3#-CM, and more prominently after TNF treatment (upper panels). Increased claudin-2 expression was also noted in the ileal crypt regions of VSL#3-treated SAMP, which recapitulates the characteristic distribution of claudin-2 in the normal, uninflamed gut [8]. Occludin expression likewise increased along the crypt-villous axis in SAMP ilea following treatment with either VSL3#-CM or TNF, most prevalently in the distal villous tips. We next examined the protein levels of occludin and claudin-2 in freshly IEC isolated from SAMP mice treated with VSL#3 by Western blot. Consistent with the aforementioned observations, occludin protein levels were increased, while claudin-2 protein levels were markedly decreased, compared to control mice (Fig. 4B). In order to relate occludin and claudin-2 changes to the observed effects on intestinal permeability, we then investigated protein expression of the two TJ proteins in the membrane and cytosolic fractions of isolated IEC preparations from SAMP intestinal loops treated with either VSL#3, TNF or vehicle. Interestingly, we observed modulation of occludin following either VSL#3 or TNF compared to vehicle control within the membrane fraction, while claudin-2 was increased in the cytosol, but not in the membrane fraction (Fig. 4C). These data suggest that occludin may play a more prominent role in modulating epithelial barrier function in SAMP mice following VSL#3 treatment.

### TNFR Expression is Essential and Contributes to the Effects of VSL#3-CM and TNF on Regulating Intestinal Barrier Function

**Figure 5:**
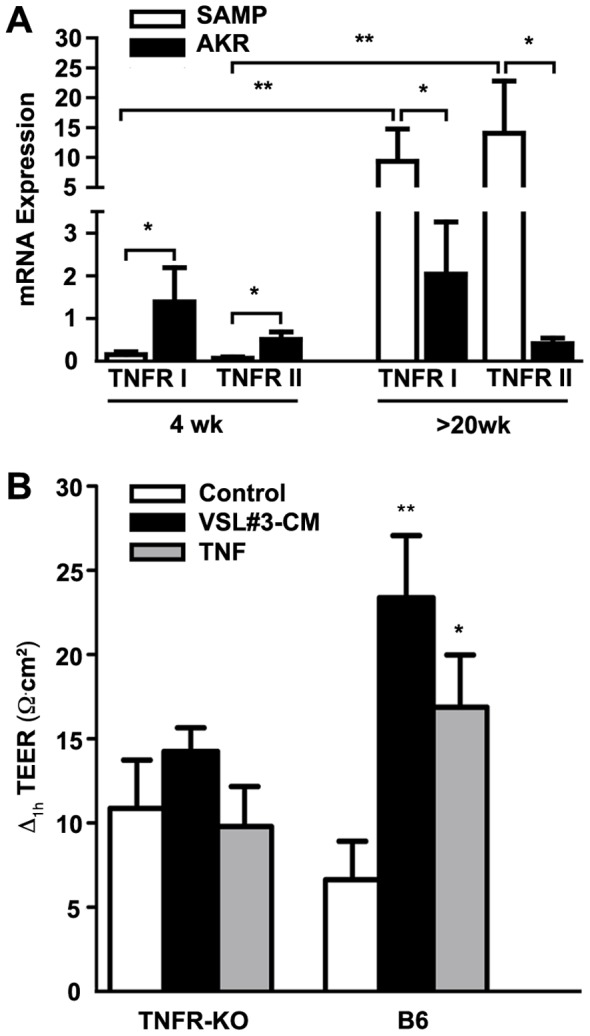
SAMP ilea differentially express TNFRs, which are essential to the effects of VSL#3-CM and TNF. (*A*), TNFRI and II mRNA expression measured by qRT-PCR was decreased in freshly isolated IEC from 4-wk-old SAMP prior to the onset of inflammation, but increased in >20-wk-old SAMP with established disease compared to age-matched AKR control mice (N = 6/exp grp). *p<0.05 and **p<0.01. (*B*), Epithelial paracellular permeability was assessed by measuring ΔTEER on *ex vivo* cultured full-thickness ilea from mice deficient in both TNFRI and TNFRII (TNFR KO) and WT controls after 1 h exposure to VSL#3-CM or TNF (N = 6/exp grp). *p<0.05 vs. WT vehicle cont.

We next investigated the expression of epithelial TNFRs in pre-inflamed and inflamed SAMP ilea, and their potential contribution to the VSL#3- and TNF-mediated effects on epithelial barrier function. TNFRI and TNFRII mRNA transcript levels were evaluated in freshly isolated IECs from 4-wk-old (pre-inflamed) or >20-wk-old (inflamed) SAMP mice (Fig. 5*A*). Although neither TNFRI nor TNFRII mRNA transcript levels changed in IEC from AKR prior to the onset of inflammation to established inflammation, mRNA expression of both TNFRs was strikingly increased in IEC from pre-inflamed compared to inflamed SAMP mice (both *p*<0.01). Interestingly, IEC derived from young, pre-inflamed SAMP displayed decreased mRNA transcripts for TNFRI and TNFRII compared to age-matched AKR (both *p*<0.05), while during established disease, mRNA expression for both TNFRs was markedly increased in SAMP IEC versus control AKR (both *p*<0.05) (Fig. 5*A*).

Finally, we explored the effects of VSL#3-CM and TNF on epithelial paracellular permeability in mice deficient in both TNFRI and TNFRII (TNFR KO mice). ΔTEER was measured in ilea after 1 h exposure to VSL#3-CM or TNF (10 ng/ml) versus vehicle control. No differences were observed in ΔTEER from TNFR KO ilea when exposed to either VSL#3-CM or TNF. In contrast, stimulation with either VSL#3-CM or TNF markedly improved (increased TEER) epithelial barrier function of WT control mice (*p*<0.05). Together, this data suggest that TNFRs are essential in mediating VSL#3- and TNF-dependent changes in intestinal epithelial paracellular permeability, and that TNFR expression on IEC may contribute to the overall effects of VSL#3 on mucosal barrier function.

## Materials and Methods

### Mice

SAMP mice were originally propagated at the University of Virginia and then at Case Western Reserve University (CWRU), with founders provided by S. Matsumoto (Yakult Central Institute for Microbiological Research, Tokyo, Japan) [9], [10]. AKR/J, as well as mice deficient in both TNFRII and II, *i.e*., B6; 29S-Tnfrsf1atm1ImxTnfr1btmImx/J (TNFR KO) and WT controls were purchased from The Jackson Laboratory (Bar Harbor, ME). All experimental mice were maintained under SPF conditions, fed standard laboratory chow (Harlan Teklad, Indianapolis, IN), and kept on 12 h light/dark cycles. All procedures were approved by the CWRU’s Institutional Animal Care and Use Committee (IACUC) (animal protocols 2009-0186 and 2011-0196) and were in accordance with the Association for Assessment and Accreditation of Laboratory Animal Care (AAALAC) guidelines.

### *Ex vivo* Permeability and Organ Culture Assays

VSL#3 (provided by Dr. Claudio De Simone, VSL#3 Pharmaceuticals, Rome, Italy) conditioned media (VSL#3-CM) was prepared as previously described [30], and diluted 1∶10 in unconditioned media for *ex vivo* permeability and organ culture assays. Transepithelial electrical resistance (TEER) assays were performed according to a previously described method [31]. In brief, distal ilea from experimental mice were removed, flushed with 0.9% NaCl, and opened longitudinally to expose mucosal surfaces. Two 3-mm squares of tissue were excised and placed apical side up on separate precut and wetted 0.4-µm pore size membranes (Costar Incorporated, Corning, NY). Specimens were mounted between two custom made Plexiglass discs with laser cut holes (2-mm diameter) to expose apical and basolateral tissue surfaces, placed into Snapwell inserts (Costar) apical side up, and fitted into a 6-well plate. 2 ml of RPMI only (control), VSL#3-CM, RPMI + TNF (10 ng/ml; Peprotech, Rocky Hill, NJ), or VSL#3-CM + a neutralizing antibody against TNF (1 µg/ml; R&D Systems, Inc., Minneapolis, MN) was added basally or 500 µl apically. TEER readings were taken using an EVOM voltmeter with an EndOhm chamber attachment (World Precision Instruments, Sarasota, FL) immediately after assembling transwell apparati and after 1 h incubation at 37°C. Baseline resistance readings were determined in transwells containing membrane inserts only and subtracted from sample values. In addition, supernatants were collected from cultured ilea exposed apically to VSL#3-CM (diluted 1∶10) and secreted cytokines measured by sandwich ELISA (eBioscience, San Diego, CA) for TNF, and by Quansys multiplex ELISA array (Quansys, Logan, UT) for IL-1β, IL-6 and IL-10. All ELISAs were performed according to manufacturers’ instructions.

### VSL#3 Administration Protocol

SAMP mice were administered high-dose VSL#3 (50×10^9^ CFU/day) mixed in their normal chow, as previously described [19]. Age-matched control mice were fed unsupplemented chow. VSL#3 was administered for 6 weeks, after which mice were killed and ilea was collected to isolate epithelial cells as described below.

### Intestinal Epithelial Cells (IEC) Isolation

Freshly isolated IECs were harvested from ileal segments of experimental mice as previously described [32], and processed for quantitative real-time PCR (qRT-PCR) or Western blot analysis as described below.

### Quantitative RT-PCR

Total RNA was isolated from homogenized tissues or from primary isolated IEC (RNeasy Mini or Miniprep Kits; Qiagen, Valencia, CA), and subsequently converted into cDNA (Tetro cDNA Synthesis Kit; Bioline, Taunton, MA). cDNA was quantified using the Applied Biosystem RT-PCR detection system and software (Applied Biosystem StepOne and StepOnePlus system, Carlsbad, CA). qRT-PCR was performed using the following primers: occludin (5′-CCCTGACCACTATGAAACAG-3′ and 5′-TTGATCTGAAGTGATAGGTG-3′), claudin-1 (5′–ACGGTCTTTGCACTTTGGT-3′ and 5′-AGTTTGCAGGATCTGGGAT-3′), claudin-2 (5′-TATCTCTGTGGTGGGCATGA-3′ and 5′CGAAGGATGCCATGAAGATT-3′), claudin-3 (5′-CCACTACCAGCAGTCGATGA-3′ and 5′-CAGCCTGTCTGTCCTCTTCC-3′), claudin-4 (5′-AGCAAACGTCCACTGTCCTT-3′ and 5′-CCCTCATCAGTCACTCAGCA), zonula occludens-1 (ZO-1) (5′-CCTAAGACCTGTAACCATCT-3′ and 5′-CTGATAGATATCTGGCTCCT-3′), and the RT^2^ Primer Assay used for TNFRI and TNFRII evaluation (SABioscience, Frederick, MD). Target mRNA was normalized to β-actin for each sample, and all samples were run in duplicate.

### *In vivo* Exposure of VSL#3 or TNF to Gut Mucosa using Ileal Loop Procedure

SAMP mice were fasted 4h prior to anesthesia via nose cone exposure to isofluorane (Baxter, Deerfield, IL) as previously described [33]. Abdomens of experimental mice were shaved and midline and subcostal incisions made through the dermis and the peritoneum. A section of ileum was isolated 0.5 cm from the cecum, a 2-cm intestinal loop fashioned, and either 100 µl of vehicle control, VSL#3-CM or TNF (10 ng/ml) was injected into the ileal loop. The peritoneum and dermis were then sutured and the animal maintained under anesthesia on a 37°C heating stage. Experimental animals were sacrificed after 2 h and ileal loops explanted and fixed as described below or used to freshly isolate IECs.

### Immunofluorescent Detection of Claudin-2 and Occludin

Paracecal regions of the intestinal ileum were explanted and fixed with 4% paraformaldehyde/PBS for 12 h followed by dehydration in 30% sucrose/PBS prior to embedding in OCT freezing media (Tissue-Tek, Sakura, Japan). 10 µm tissue sections were cut on a CM3050s cryostat (Leica, Buffalo Grove, IL) and mounted on Superfrost Plus slides (VWR, Batavia, IL). Tissue sections were permeabilized and blocked in PBS containing 0.3% Triton X-100 (Sigma-Aldrich, Milwaukee, WI) and 10% goat serum (Jackson Immunoresearch, West Grove, PA), followed by staining with primary antibodies for claudin-2 (1∶300) or occludin (1∶100), and an Alexa Fluor-conjugated secondary antibody (1∶500). TJ proteins were then detected and immunolocalized using Prolong Gold or Prolong Gold plus DAPI (all from Invitrogen, Carlsbad, CA). 3D image stacks were acquired by confocal microscopy (ZEISS LSM 510, Thornwood, NY), and images displayed as 2D maximum intensity projections.

### Subcellular IEC Fractionation

Freshly isolated IECs were harvested from ileal segments of experimental mice in 10 mM HEPES, pH 7.4, 100 mM KCl, 3mM NaCl, 1 mM Na_2_ATP, 3.5 MgCl_2_, 1 mM PMSF, Protease Inhibitor Cocktail and Phosphatase Inhibitor Cocktail (Sigma-Aldrich, Milwaukee, WI). Cell lysis was achieved using cell douncers (Fischer Scientific, Pittsburgh, PA), and lysates were cleared of nuclear material by 1000 x g centrifugation for 10 min at 4°C. Supernatants were then centrifuged (100,000 x g) for 1 h at 4°C to separate membrane from cytosolic fractions. Resulting supernatants were collected as cytosolic fractions and pellets (membrane fractions) were resuspended in RIPA buffer (Fischer Scientific, Pittsburgh, PA) with 1% OG (n-Octyl-β-D-Glucopyranoside) and then sonicated 3x (70% duty cycle) on ice.

### Western Blot Analysis

Western blot analysis was performed on total protein extracts or cellular fractions of freshly isolated IECs from experimental mice. Equal amounts of protein were separated by SDS-PAGE and transferred onto polyvinylidene fluoride membranes. Membranes were blocked for 1 h with 5% wt/vol dry milk in Tris-buffered saline containing 0.1% Tween-20, and incubated with primary antibodies (Invitrogen, Carlsbad, CA) in blocking buffer overnight at 4°C, followed by incubation with HRP-conjugated secondary antibody (Santa Cruz Biotechnology, Santa Cruz, CA). Proteins were visualized using ECL detection system (GE Healthcare Biosciences, Pittsburgh, PA).

### Statistical Analysis

Statistical analyses were performed using GraphPad Prism Version 5 (GraphPad Software, San Diego, CA). Differences were evaluated by one-way ANOVA Kruskal-Wallis test with Dunns posthoc test and a *p* value of <0.05 was considered to be significant; *p* value of <0.01 was indicated as **.

## Discussion

In the present study, we addressed the potential mechanism of action of the probiotic mixture, VSL#3, for its ability to “boost” innate mucosal immune responses by inducing TNF production, particularly during early phases of gut inflammation, in the SAMP mouse model of CD-like ileitis [19]. Specifically, we showed that VSL#3-CM directly stimulates TNF, as well as other innate-type cytokines, when exogenously applied to *ex vivo* cultured pre-inflamed ileal tissues from SAMP mice. These effects were associated with decreased paracellular permeability and modulation of the epithelial TJ proteins, claudin-2 and occludin, which are inherently dysregulated in SAMP mice [7]. Interestingly, the effects of VSL#3-CM were reproduced by stimulation with exogenously applied recombinant TNF, and abrogated by blockade with a neutralizing antibody against murine TNF, confirming a central and direct role of TNF in this process. These finding are consistent with our previous study showing that the global effect of VSL#3-induced TNF results in the downstream prevention of ileitis in young, uninflamed SAMP mice and improved *in vivo* barrier function, whereas SAMP with established disease did not benefit therapeutically from VSL#3 treatment [19].

The results of our studies show that VSL#3-CM stimulates TNF production and generates permeability changes in SAMP and C57BL/6, but not in AKR ilea, suggesting that AKR mice inherently do not respond to VSL#3. Since SAMP mice are on a mixed background [12] and contain genes from both C57BL/6 and AKR strains, we speculate that the response to VSL#3 and TNF in SAMP mice is likely dependent on the contribution of C57BL/6 genes. Our laboratory is currently investigating this hypothesis using SAMP consomic mice with specific C57BL/6 chromosomal replacement. The protective effects of TNF in this model were somewhat surprising considering the established proinflammatory role of TNF in the pathogenesis of chronic intestinal inflammation and IBD. However, increasing evidence suggests that TNF possesses “paradoxical” effects that are dependent on the phase of disease progression and the presence of TNFR-bearing effector cells. TNF is a classic proinflammatory cytokine that is well known to be critically involved in the pathogenesis of CD, as evidenced by the beneficial effects following treatment with anti-TNF antibodies (*e.g*., infliximab) [34]. However, recent studies have highlighted the anti-inflammatory properties of TNF for various cell types [32], particularly in the gut, and its role in protecting and maintaining the integrity of the intestinal epithelium [35]. In the present study, epithelial permeability was restored in ileal tissues from young, pre-inflamed SAMP mice following exposure to VSL#3-CM, and reversed upon treatment with a neutralizing antibody against TNF. Furthermore, we previously demonstrated that VSL#3 not only promotes epithelial TNF production, but also stimulates NF-kB activation in IEC [19]. As such, VSL#3-dependent NF-kB regulation in IEC may play a key role in epithelial barrier function and protection. In support of this concept, deletion of the NF-kB activator, IkB kinase β (IKKβ), specifically in IEC (IKKβ^IEC−/−^), does not prevent dextran sodium sulfate (DSS)-induced colitis [36], and mice selectively defective in IEC for the main IKK subunit, IKKγ (IKKγ^IEC−/−^), spontaneously develop colitis [37].

The protective effects of TNF during acute phases of gut inflammation are further supported by a study reporting that TNF deletion in mice leads to increased severity and exacerbation of disease in acute DSS-induced colitis [38]. Moreover, TNFRI-mediated activation of innate immunity has been shown to play an important role in preventing intestinal damage-associated mortality [39] and is protective in the trinitrobenzene sulfonic acid mouse model of colitis [40], [41]. More recently, TNF was shown to suppress acute intestinal inflammation though a mechanism that involves local glucocorticoid synthesis within the gut mucosa [42]. Since DSS-induced colitis is partially mediated by IEC apoptosis, an alternative hypothesis is that TNF inhibits IEC apoptosis, thus improving overall intestinal barrier function. Further studies are warranted to establish the validity of this hypothesis. Therefore, altogether these results suggest that TNF may possess protective, homeostatic activities, particularly in the GI tract, which may be specific for early, acute phases of inflammation. During this time, the first, initial response of the mucosal environment may be to enhance innate immunity against luminal antigens through the production of innate-type cytokines in an attempt to return the gut to a state of mucosal homeostasis. Defects or dysregulation in the host’s ability to mount appropriate, early innate immune responses may therefore result in the initiation and perpetuation of chronic intestinal inflammation. In addition, the possibility exists that during chronic, established ileitis, the effects of probiotics, and induced epithelial TNF, are not effective due to either the presence of copious amounts of macrophage-derived TNF, different TNFR-bearing effector cell populations during later, chronic phases of intestinal inflammation, or actual downregulation of TNFRs, likely as a negative feedback mechanism in response to increasing local levels of TNF.

Abnormalities in SAMP small intestinal barrier function are accompanied, as previously reported [8], by alterations in TJ proteins with an overexpression of claudin-2 and decreased expression of occludin. In this context, several studies have shown that increased claudin-2 and decreased occludin expression in epithelial TJs result in barrier dysfunction [13]–[16]. For example, overexpression of claudin-2 in MDCK I cells leads to increased epithelial paracellular permeability due to aberrant claudin-2/claudin-2 pairing on adjacent cells compared to pairings with other claudin (1–4) proteins [15]. Conversely, overexpression of occludin in MDCK cells results in an increased number and width of TJ connections and a decrease in epithelial paracellular permeability, indicative of a tighter barrier [14]. These aforementioned trends in claudin-2 and occludin expression have also been reported in patients with both CD and UC [17], [18], [43]. Nevertheless, in claudin-2 and occludin deficient mice, structure and function of TJ proteins in the small intestine are indistinguishable from those of wild type control mice [44]. However, these results deserve careful consideration since compensatory mechanisms may exist in genetically-manipulated mice that underestimate the physiological relevance of these molecules. One of the most interesting findings of our study is the ability of VSL#3-CM and TNF to decrease *ex vivo* ileal paracellular permeability and to regulate the expression of epithelial TJ proteins. Specifically, upregulation of occludin in the insoluble (membrane) fraction of IEC isolated from SAMP treated with VSL#3 suggests that occludin may function as the prominent TJ protein regulating barrier function in response to probiotic treatment. In fact, one of the beneficial roles of probiotics is to promote intestinal barrier integrity by influencing the structure of TJ proteins [45]. In addition, *in vivo* studies have demonstrated the global protective effects of probiotics on the gut epithelium in different animal models of intestinal inflammation [46]–[48]. In particular, VSL#3 has been shown to improve intestinal barrier function in DSS-induced colitis by preventing the reduction and redistribution of TJ proteins, specifically augmenting the expression of ZO-1, and by inhibiting increases in the apoptotic ratio [45]. Thus, the protective effects of TNF-induced VSL#3 treatment in SAMP mice could be partially attributed to inhibition of IEC apoptosis. Further studies are warranted in order to fully address this mechanism. Moreover, *in vitro* treatment of epithelial cells with probiotics, or metabolites secreted by probiotics, leads to an increase in ZO-1 and occludin expression, while decreasing claudin-2 [46]; this study also demonstrated the ability of probiotics, or probiotic by-products, to either directly or indirectly modulate epithelial paracellular permeability and control ion selectivity of TJs [46].

Epithelial TJ proteins represent the principal determinants of intestinal paracellular permeability and, although mechanism(s) leading to changes in epithelial TJ protein expression have been extensively investigated, it remains to be proven what specific conditions lead to alterations in particular TJ proteins and overall TJ disruption. In fact, much work has focused on cytokine-dependent regulation of the TJ complex. In this context, a direct role of TNF has been implicated in causing loss of barrier function in cultured intestinal epithelial cell monolayers [49], [50]. In addition, treatment with infliximab (anti-TNF) has been reported to restore barrier function in CD, and some UC, patients [51]–[53]. However, conflicting studies exist regarding the interpretation of the effects of TNF that may reflect differences in the specific epithelial cell line used or variations in the dose and length of the treatment with TNF [54]. For example, in the intestinal epithelial cell line, Caco-2, TNF stimulation results in a delayed effect on permeability by increasing small molecule flux within 24 hrs, but reduction in TEER is not observed until 48 hrs post treatment [55]. IFNγ also represents a proinflammatory cytokine found at elevated levels in the intestinal mucosa of IBD patients, that in addition to its immunomodulatory role during chronic inflammation, acts directly to regulate epithelial and endothelial barrier function [56]–[58]. Interestingly, a recent report shows that IFNγ exacerbates intestinal inflammation by distinct temporal regulation of converging β-catenin signaling pathways, and this effect is potentiated by TNF, which synergistically cooperates to promote cell proliferation and thereby, potential healing. Conversely however, extended exposure to these cytokines appears to inhibit epithelial cell proliferation and apoptosis [59]. As such, temporal (phase and length) and quantitative differences in the expression patterns of particular proinflammatory mediators, as well as their specific receptors, may influence mechanistic diversity in TJ protein expression and function, and likely reflects changes in overall barrier integrity during acute versus chronic phases of gut inflammation. Our results demonstrate a central role of TNF in ameliorating paracellular permeability during early phases of ileitis in a spontaneous model of CD-like ileitis that closely resembles the human condition.

Our studies have significant and translational implications since it is well known that up to 50% of CD patients do not respond to anti-TNF treatment modalities [60]. Interestingly, whether anti-TNF therapy exacerbates disease activity in patients classified as “non-responders” is not routinely evaluated in clinical practice. Based on the results of our studies, we speculate that individuals who fall into this “non-responder” patient population may be in a disease state/phase that depends on the anti-inflammatory properties of TNF, rather than its recognized proinflammatory activity. As such, it would interesting to evaluate whether “boosting” the innate immune system by using probiotic therapy would produce beneficial effects in this patient population.

Taken together, our study further support the hypothesis that CD may be initiated by a deficit in intestinal innate immunity, rather than an overly aggressive adaptive immune response to luminal antigens in which innate-type cytokines, such as TNF, play a critical role [61]. This working hypothesis, and the role the probiotic formulation, VSL#3, may play in improving epithelial barrier function and subsequent gut inflammation, in ileitis-prone SAMP mice is summarized in Fig. S1. In this context, “boosting” the intestinal innate immune system with immunostimulatory agents, such as probiotics, and before the onset of disease, may represent a novel therapeutic modality to prevent or induce a state of permanent remission in patients with CD.

## Supporting Information

Figure S1 **Working hypothesis of TNF-dependent probiotic modulation of epithelial barrier function during the early phases inflammation.** One of the earliest features characteristic of ileitis-prone SAMP mice are epithelial alterations, including global changes in epithelial architecture, expansion of villi, as well as Paneth and goblet cell hyperplasia. Interestingly, an inherent increase in small intestinal epithelial paracellular permeability is also present in young SAMP, prior to the onset of inflammation (*left*). Upon treatment of SAMP mice with VSL#3 during the early phases of inflammation, “boosting” of innate-type responses occurs, which include production of IEC-derived TNF and other early innate cytokines, including IL-1 and IL-6, as well as the anti-inflammatory cytokine, IL-10. In addition, VSL#3-induced TNF decreases ileal epithelial paracellular permeability by modulating the TJ proteins, claudin-2 (decrease) and occludin (increase), with the net effect of improving overall epithelial barrier function (*right*).
(TIFF)
