# Supplementary figures and images for: Probiotic Bacteria Regulate Intestinal Epithelial Permeability in Experimental Ileitis by a TNF-Dependent Mechanism
Source: PLoS One. 2012 Jul 25;7(7):e42067. doi: 10.1371/journal.pone.0042067 (PMC3405026; doi:10.1371/journal.pone.0042067)

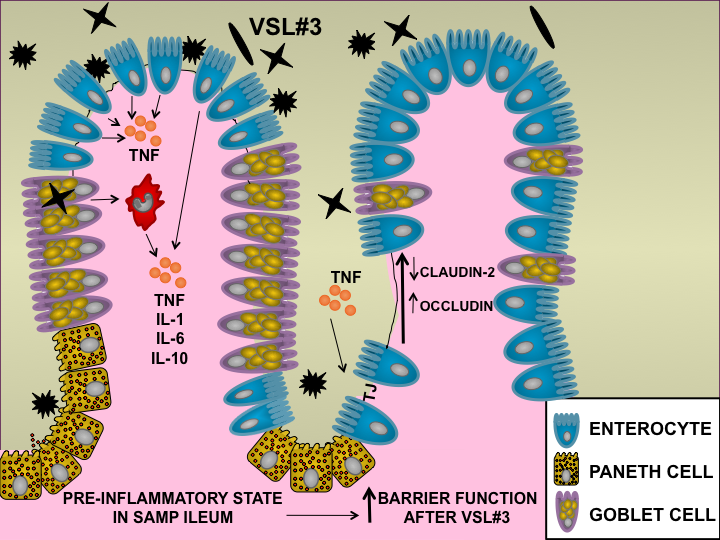

Supplement: Figure S1 — Working hypothesis of TNF-dependent probiotic modulation of epithelial barrier function during the early phases inflammation. One of the earliest features characteristic of ileitis-prone SAMP mice are epithelial alterations, including global changes in epithelial architecture, expansion of villi, as well as Paneth and goblet cell hyperplasia. Interestingly, an inherent increase in small intestinal epithelial paracellular permeability is also present in young SAMP, prior to the onset of inflammation (left). Upon treatment of SAMP mice with VSL#3 during the early phases of inflammation, “boosting” of innate-type responses occurs, which include production of IEC-derived TNF and other early innate cytokines, including IL-1 and IL-6, as well as the anti-inflammatory cytokine, IL-10. In addition, VSL#3-induced TNF decreases ileal epithelial paracellular permeability by modulating the TJ proteins, claudin-2 (decrease) and occludin (increase), with the net effect of improving overall epithelial barrier function (right). (TIFF) [file pone.0042067.s001.tiff]
